# Gold Nanoparticle-Mediated DNA Damage Under FLASH Electron-Beam Irradiation: A Monte Carlo Study

**DOI:** 10.3390/nano16171116

**Published:** 2026-09-04

**Authors:** Chloe Doen Kim, James C. L. Chow

**Affiliations:** 1 Radiation Medicine Program, Princess Margaret Cancer Centre, University Health Network, Toronto, ON M5G 1X6, Canada; 2 Department of Radiation Oncology, University of Toronto, Toronto, ON M5T 1P5, Canada; 3 Department of Materials Science and Engineering, University of Toronto, Toronto, ON M5S 3E4, Canada

**Keywords:** gold nanoparticles, FLASH radiotherapy, ultrahigh-dose-rate irradiation, radiosensitization, DNA damage, reactive oxygen species, electron beams, Geant4-DNA, Monte Carlo simulation, nanodosimetry

## Abstract

FLASH radiotherapy, which delivers radiation at ultrahigh dose rates (UHDRs) exceeding 40 Gy/s, has attracted considerable attention because of its potential to spare normal tissues while maintaining tumour control. Gold nanoparticles (GNPs) are promising radiosensitizers that enhance radiation-induced biological effects through increased production of reactive oxygen species (ROS) and subsequent DNA damage. However, the influence of GNPs on DNA damage under FLASH irradiation remains poorly understood. In this study, Geant4-DNA Monte Carlo simulations were performed to investigate the effects of GNP size and dose rate on DNA damage during UHDR electron-beam irradiation. DNA damage was quantified through both direct and indirect mechanisms based on energy deposition in DNA backbone segments and interactions between radiation-induced radical species and DNA, and was evaluated relative to equivalent water nanoparticle (WNP) controls. The results demonstrated a dose rate-dependent reduction in both relative single-strand breaks (RSSBs) and relative double-strand breaks (RDSBs) arising from direct and indirect DNA damage mechanisms. For the 10 keV monoenergetic model condition, the 10 nm GNP produced the greatest radiosensitization among the particle sizes investigated, with the matched GNP/WNP analysis showing a maximum 5-fold enhancement in direct SSB yields. In contrast, substantially weaker GNP-specific enhancement and no comparable size-dependent effect were observed at 1 MeV. These findings demonstrate that GNP-mediated radiosensitization depends on both nanoparticle size and electron energy under the irradiation conditions investigated. The greater enhancement observed for the 10 nm GNP at 10 keV should therefore not be interpreted as identifying a universally optimal GNP size for FLASH radiotherapy.

## 1. Introduction

Cancer continues to be one of the challenges in global health and is among the leading causes of death worldwide, accounting for nearly one in six deaths [1]. Despite substantial progress in cancer prevention and treatment, it was reported in 2022 that one in five people was estimated to develop cancer during their lifetime, while approximately one in nine men and one in twelve women were expected to die from the disease [2].

Although radiotherapy, surgery and chemotherapy have been widely used to treat cancer patients, both individually and multimodally [3,4], radiotherapy remains a cornerstone in the treatment of various malignancies, delivering to around 40% of patients who survive at least 5 years [4]. Photon and electron beams generated by a medical linear accelerator (LINAC), the device most used for external beam radiotherapy, deposit ionizing energy (referred to as the dose) in cells as they pass through the human body, leading to cellular apoptosis or genetic mutations [5,6]. Photons interacting with electrons bound to atoms can cause the photoelectric effect, resulting in the ejection of photoelectrons. In contrast, interactions with free electrons lead to Compton scattering, producing a scattered photon and a recoil electron. Electrons generated by the photoelectric effect and Compton scattering deposit most of their energy in cells, leading to DNA damage [7]. Recent advances in modern radiation delivery techniques, such as volumetric modulated arc therapy (VMAT), can deliver photon beams with highly conformal dose distributions, improving target volume coverage while minimizing damage to healthy tissues [8]. In addition, image-guided radiotherapy (IGRT) enables real-time assessment of tumour position and size during treatment using imaging modalities such as MRI. This capability allows for precise delivery of radiation to the tumour while decreasing exposure to surrounding normal tissues. Consequently, IGRT is associated with a reduced risk of radiation-induced toxicity and facilitates the development of shorter and more efficient radiation treatment regimens [9]. Nevertheless, the radiation dose necessary for local tumour control remains constrained by toxicity to surrounding normal tissues and the priority of reducing the treatment-related side effects [10].

FLASH radiotherapy (FLASH-RT), which involves the delivery of radiation at ultrahigh dose rates (UHDRs) exceeding 40 Gy/s, is a new technique aimed at enhancing treatment efficacy while sparing healthy tissues [11,12,13]. Multiple hypotheses have been proposed to explain the mechanisms of the FLASH effect. In the transient oxygen depletion hypothesis, a temporary hypoxic state in normal tissues during UHDR irradiation induces increased resistance to radiation damage [14,15]. The experimental investigations have demonstrated a sparing effect in vitro [16,17,18,19] and in vivo [20,21].

In another theory, tumour tissues are hypothesized to exhibit responses to reactive oxygen species (ROS) that are distinct from those of normal tissues. Cancer cells maintain elevated steady-state ROS levels, which contribute to increased oxidative damage following exposure to ionizing radiation [22]. In contrast, normal cells exhibit greater resistance to ROS due to their robust antioxidant defence systems [23,24]. The limited diffusion of ROS produced under UHDR conditions may partially contribute to the selective targeting of cancer cells as well [23]. In addition, a protective immune response under FLASH-RT is also hypothesized. In contrast to conventional radiotherapy (CONV-RT), FLASH-RT may reduce inflammation in normal tissues while enhancing anti-tumour immunity, preserving a greater number of circulating immune cells [25,26].

Theoretical simulation studies [27,28,29] and preclinical data with various radiation modalities [21,30,31,32] have demonstrated that FLASH-RT provides excellent disease control with minimal or no side effects at high dose rates. Furthermore, clinical trials related to UHDR-RT have been conducted worldwide. The tumour control rate of UHDR (166 Gy/second) electron-beam RT for a patient with cutaneous lymphoma was comparable to that of CONV-RT (0.08 Gy/second), and there was no difference in acute reactions or late effects at 2 years [33]. In a clinical trial of 10 patients with painful extremity bone metastases, proton FLASH radiotherapy (8 Gy delivered in a single fraction at a dose rate of 60 Gy/s) showed treatment efficacy and an adverse event profile comparable to CONV-RT [34]. The remarkable capacity for normal tissue protection, alongside therapeutic efficacy on par with CONV-RT, demonstrates the clinical feasibility of FLASH-RT.

Concurrently, gold nanoparticles (GNPs), one of the engineered nanomaterials (ENMs) with at least one dimension between 1 and 100 nm as defined by the International Organization for Standardization (ISO) [35,36], have gained significant attention as radiosensitizers in the field of radiation oncology due to their high X-ray absorption and unique physicochemical properties [37,38,39]. The large surface-to-volume ratio, excellent biocompatibility, low toxicity, ease of surface modification, and high atomic number (Z = 79) of GNPs, which enhances radiation absorption, make them particularly attractive in bionanotechnology [37,40,41].

Many studies have demonstrated that GNPs enhance the biological effects of radiation through both physical and chemical mechanisms. They increase the physical dose delivered to the tumour and simultaneously interfere with the biological mechanisms that enable tumour cells to recover from radiation-induced damage [42]. Additionally, GNPs increase radiosensitivity by stimulating ROS production, which drives oxidative stress and subsequent cellular and DNA damage in cancer cells [43,44,45,46,47,48,49,50]. In particular, the photoelectric effect directly results in the emission of primary photoelectrons and triggers a cascade of Auger electrons. As these Auger electrons propagate through GNPs, they undergo inelastic scattering, lose energy, and generate successive generations of lower-energy secondary electrons [51]. With their limited penetration in water, low-energy electrons deposit energy densely along short tracks, inducing water radiolysis and the formation of ROS [52].

DNA strand breaks are classified as single-strand breaks (SSBs) or double-strand breaks (DSBs). In SSBs, the intact antiparallel strand preserves the physical integrity and genetic information needed to guide accurate repair of the damaged strand. In contrast, DSBs involve breaks in both strands of the DNA helix, resulting in the loss of physical continuity and genetic information at the break site [53]. Ionizing radiation induces DSBs through direct radiation energy deposition to the sugar backbone of DNA, but ROS induced near GNPs can also indirectly damage DNA in cancer cells [54]. By amplifying ROS-induced DNA damage, GNPs introduce new mechanistic insights and strategic opportunities for improving the therapeutic efficacy of radiotherapy [55].

While we recently reported that small GNPs (5–10 nm) enhanced ROS production under 1 MeV electron-beam irradiation at UHDR of 60 Gy/s [56], the synergistic impact of GNP-induced ROS on DNA damage under FLASH-RT conditions remains largely unexplored. Monte Carlo (MC) simulation is widely regarded as a fundamental tool for studying clinical dosimetry in megavoltage electron and photon beams [57]. In particular, Geant4-DNA, an open-source developed by the European Organization for Nuclear Research (CERN), provides detailed interaction-by-interaction transport modelling of electrons, protons, hydrogen atoms, alpha particles, and selected ions in liquid water at nanometre resolution [58]. In Geant4-DNA, water radiolysis is modelled as a three-stage process: physical, physico-chemical, and chemical. It is specifically designed to simulate ionizing radiation interactions with simplified geometrical structures of biological targets [27].

Previous MC studies involving GNPs have largely focused on conventional dose rates, with limited consideration of the unique characteristics associated with UHDR delivery [59,60,61,62,63]. To the best of our knowledge, few simulation studies have explored the combined effects of GNPs and FLASH-RT on DNA damage, particularly regarding ROS generation during water radiolysis under UHDR conditions. In this study, we address this gap, employing Geant4-DNA MC simulations to investigate the impact of GNPs on DNA damage under UHDR electron-beam irradiation. DNA damage was assessed through both direct and indirect mechanisms, based on energy deposition in DNA backbone segments and stochastic interactions between radical species and the DNA backbone. It was evaluated relative to an equivalent water nanoparticle (WNP) control as a function of GNP size and dose rate.

## 2. Materials and Methods

### 2.1. Simulation Geometry and Configuration Using Geant4-DNA

MC simulations were conducted using Geant4-DNA, an extension of the general-purpose Geant4 toolkit developed by CERN. It is designed to simulate the physical, physicochemical, and chemical interactions of ionizing radiation with high-Z (high atomic number) materials at nanometric spatial resolutions and to assess their impact on cellular function and survival probability following irradiation [64,65]. In this study, MC simulations were performed using Geant4 version 11.2. This version features a comprehensive physics model that simulates the full sequence of radiation-induced interactions, from energy deposition and track structure formation to the evolution of chemical species for the surrounding water medium. The updated chemistry module further enables time-resolved radiolysis simulations, providing detailed insight into the spatiotemporal dynamics of reactive oxygen species generated by irradiation [63]. Particle interactions within the gold nanoparticle (GNP) were modelled using the Livermore electromagnetic physics model because of its proven accuracy in simulating low-energy electromagnetic interactions in high-atomic-number (high-Z) materials.

The simulations were conducted to investigate the response of a GNP embedded in an aqueous medium under UHDR electron-beam irradiation. The framework was designed to characterize both the physical and chemical stages of radiation interaction, enabling the quantification of energy deposition and the subsequent production of ROS in the nanoparticle microenvironment, resulting in the formation of early species (e.g., H_3_O+, e−aq, OH•, H•, and H_2_O_2_). The resulting species are subsequently tracked for up to 10 ns to account for their diffusion and chemical interactions in the aqueous environment. After energy deposition by primary and secondary electrons, the simulation proceeds through physicochemical processes, such as ionization, excitation, and dissociation, leading to the formation of the early species. The resulting species are subsequently tracked for up to 10 ns to account for their diffusion and chemical interactions in the aqueous environment. ROS yield was tracked at this time point, as previous studies have shown that radical interactions are most biologically significant within this timeframe [66,67].

A spherical water phantom with a radius of 2 μm was employed to model the biological environment. The phantom size was selected to be sufficiently large to prevent the escape of secondary electrons and reactive oxygen species (ROS) from the simulation volume, while remaining small enough to ensure computational efficiency [68]. The DNA model was generated using the DNAFabric tool (version from 2019) [69]. The software is designed to build the complete genomic content of human cells within a three-dimensional nuclear geometry, representing the continuous chromatin fibre at single-base-pair (bp) resolution [70]. The DNA model was positioned at the centre of the 2 µm radius sphere. The model consisted of a 40 nm piece of heterochromatin fibre containing 3640 nucleotide pairs [67], with the entire DNA structure centrally embedded within the sphere.

**Figure 1:**
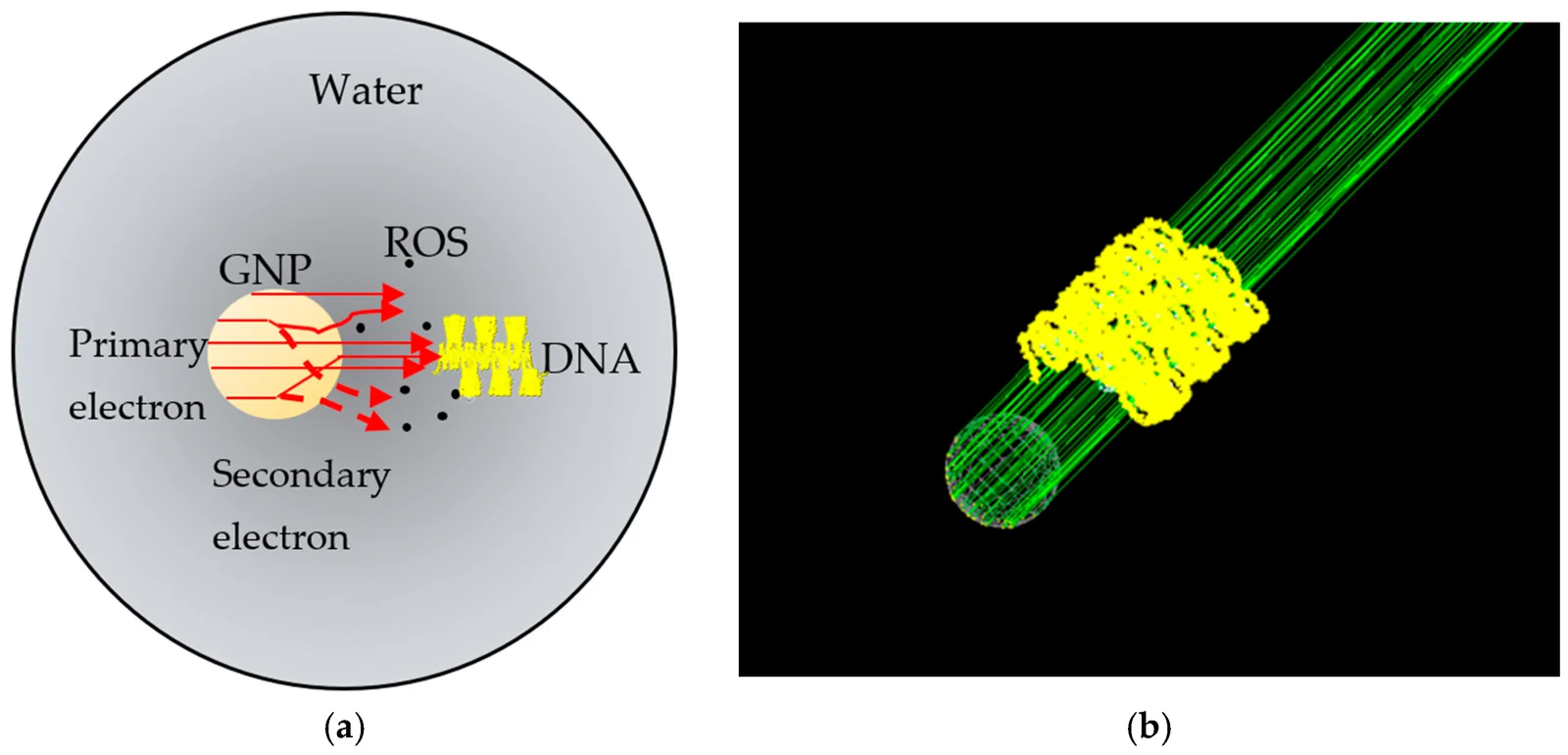
MC simulation setup using Geant4-DNA. (**a**) A GNP (light orange), with a diameter of 5, 10, 25, or 50 nm, is positioned at a fixed distance of 10 nm from the DNA model (yellow). The GNP and DNA model are positioned at the centre of a water sphere with a radius of 2 μm. ROS, incident electrons of 10 keV or 1 MeV, and secondary electrons are denoted by black dots, solid red lines, and dashed red lines, respectively. (**b**) The experimental setup in Geant4-DNA consists of the spherical water phantom (radius of 2 µm) embedded with the GNP (grey) of varying size, DNA model (yellow), and electron beams emitted from the surface of the GNP.

A GNP with a diameter of 5, 10, 25, or 50 nm was placed at a fixed distance of 10 nm from the DNA model to investigate the size-dependent effects of GNPs on DNA strand breakage. As shown in Figure 1a, electron beams (red solid lines) with energies of 10 keV and 1 MeV were emitted from the surface of the GNP hemisphere opposite the DNA model and directed toward the DNA model. Secondary electrons (red dashed lines) generated from interactions within the GNP, together with ROS (black dots), are also illustrated. For each primary electron, the emission position was randomly sampled over the GNP hemisphere surface. The experimental setup in Geant4-DNA is shown in Figure 1b, with the spherical water phantom (radius of 2 µm) embedded with the GNP and DNA model.

Two monoenergetic electron energies, 10 keV and 1 MeV, were selected as model conditions to investigate the energy dependence of GNP-mediated DNA damage at the nanoscale. In particular, the 10 keV condition was used to provide mechanistic insight into the interactions of low-energy electrons with GNPs, including secondary-electron production, local energy deposition, and subsequent ROS-mediated DNA damage. This monoenergetic 10 keV condition is not intended to directly represent a clinical FLASH electron beam, which consists of a broad electron energy spectrum. Rather, it represents a simplified low-energy model condition relevant to the electron-energy degradation and secondary-electron processes that occur as clinical electron beams traverse biological tissue. The 1 MeV condition was included as a higher-energy comparison to investigate the dependence of GNP-mediated radiosensitization on electron energy [56].

### 2.2. MC Simulation of UHDR Electron-Beam Irradiation Condition

The characteristics of the Oriatron eRT6 (PMB, Rousset, France), an experimental high-dose-per-pulse linear accelerator specifically designed to deliver electron beams at variable dose rates [71], were incorporated into the MC simulation model developed for UHDR electron-beam irradiation. The LINAC can deliver doses of up to 10 Gy per pulse with a 2 µs pulse duration at a repetition rate of 100 Hz. To achieve realistic MC simulations, this study simulated a single radiation pulse, following the methodology proposed by Chappuis et al. [68]. Specifically, the total number of electrons emitted from the GNP surface was adjusted so that the energy deposited by the traversing electrons within the water sphere corresponded to absorbed doses of 0.4, 0.6, 1.0, 1.5, 2.0 and 3.0 Gy per pulse. Accordingly, the number of primary electrons simulated was determined separately for each irradiation condition by the energy deposition required to reproduce the prescribed dose per pulse, rather than by using a fixed number of particle histories across all conditions. The selected dose-per-pulse conditions corresponded to average dose rates of 40, 60, 100, 150, 200 and 300 Gy/s, respectively. These dose rates fall within the UHDR regime and satisfy the criteria for FLASH radiotherapy.

### 2.3. DNA Damage Quantification

Among the various ROS, short-lived radicals, particularly hydroxyl radicals (OH•), are considered the primary mediators of oxidative biological damage owing to their exceptionally high reactivity. These radicals play a pivotal role in inducing DNA DSBs, one of the most deleterious forms of DNA damage [72]. Since OH• radicals have a short lifetime (approximately 10 ns) [73], DNA damage was quantified at 10 ns in the simulations to capture the effects of OH• radical-induced damage.

DNA damage is generally classified into direct and indirect damage. Direct damage occurs when the incident radiation beam deposits energy directly into the DNA molecule, resulting in physical damage to the DNA structure. In contrast, indirect damage is caused by the interaction between DNA and reactive free radicals, particularly OH•, generated through the radiolysis of water. Previous studies have reported that a DNA SSB induced by direct damage occurs when 17.5 eV or more of energy is deposited within a DNA backbone segment. For indirect DNA damage, each OH• interaction with the DNA backbone is modelled as producing a SSB with a probability of 40% [74,75]. In addition to SSBs, a DNA DSB is scored when two SSBs occur on opposite DNA strands within 10 base pairs.

To quantify the radiosensitizing effect of GNPs on DNA SSBs and DSBs under UHDR irradiation, a control simulation was performed by replacing the GNP with a WNP of identical size and at the same location, while keeping all other simulation parameters and the geometry unchanged. For each electron energy, the relative SSB (RSSB) and DSB (RDSB) yields per Gy were calculated as the ratio of the SSB or DSB count for each tested condition to the SSB or DSB count observed with WNP of 5 nm at the lowest dose rate (40 Gy/s) in Equation (1). RSSBs and RDSBs enable the independent evaluation of the impact of GNP size and UHDR on SSB and DSB yields relative to the reference conditions of a 5 nm WNP and a dose rate of 40 Gy/s, respectively.

(1) $$RSSB\;or\;RDSB=\frac{SSB\;or\;DSB\;count\;of\;a\;simulated\;condition}{SSB\;or\;DSB\;count\;with\;5\;nm\;WNP\;at\;dose\;rate\;of\;40\;Gy/s}$$

The RSSB and RDSB metrics normalized to the 5 nm WNP at 40 Gy/s were retained to characterize variations in DNA damage yields with nanoparticle size and dose rate using a common reference condition. However, because baseline SSB and DSB yields may differ among WNP sizes and irradiation conditions, these normalized quantities alone do not provide a direct measure of the radiosensitization attributable specifically to the GNP.

Therefore, to isolate the GNP-specific radiosensitization effect, an enhancement ratio was additionally calculated by comparing each GNP with its corresponding WNP control of identical size under the same electron energy and dose-rate condition. The enhancement ratios for SSB, Equation (2) and DSB, Equation (3) yields were defined as:

(2) $$SSB\;Enhancement\;Ratio=\frac{SSB\;yield\;with\;GNP}{SSB\;yield\;with\;matched\;WNP}$$

and

(3) $$DSB\;Enhancement\;Ratio=\frac{DSB\;yield\;with\;GNP}{DSB\;yield\;with\;matched\;WNP}$$

where the numerator and denominator represent the SSB or DSB yields obtained with the GNP and the corresponding same-sized WNP, respectively, under identical electron energy and dose-rate conditions. An enhancement ratio greater than 1 indicates increased DNA damage in the presence of the GNP relative to the matched WNP control, whereas a ratio below 1 indicates a reduction. This matched-control analysis provides a direct measure of GNP-specific radiosensitization while accounting for differences in the baseline DNA damage yields among WNP sizes and irradiation conditions.

A single Monte Carlo simulation realization was performed for each combination of nanoparticle type, nanoparticle size, electron energy, and dose-rate condition. Consequently, replicate-based statistical quantities such as the standard deviation, standard error, and confidence intervals could not be determined from the present simulations. No formal statistical hypothesis testing was performed. Therefore, comparisons among simulation conditions are interpreted descriptively based on the calculated SSB and DSB yields, normalized RSSB/RDSB values, and matched GNP/WNP enhancement ratios. Terms implying statistical significance were avoided when describing comparisons among the present simulation results.

## 3. Results

### 3.1. Influence of GNP Radiosensitization on RSSBs Under UHDR Irradiation

**Figure 2:**
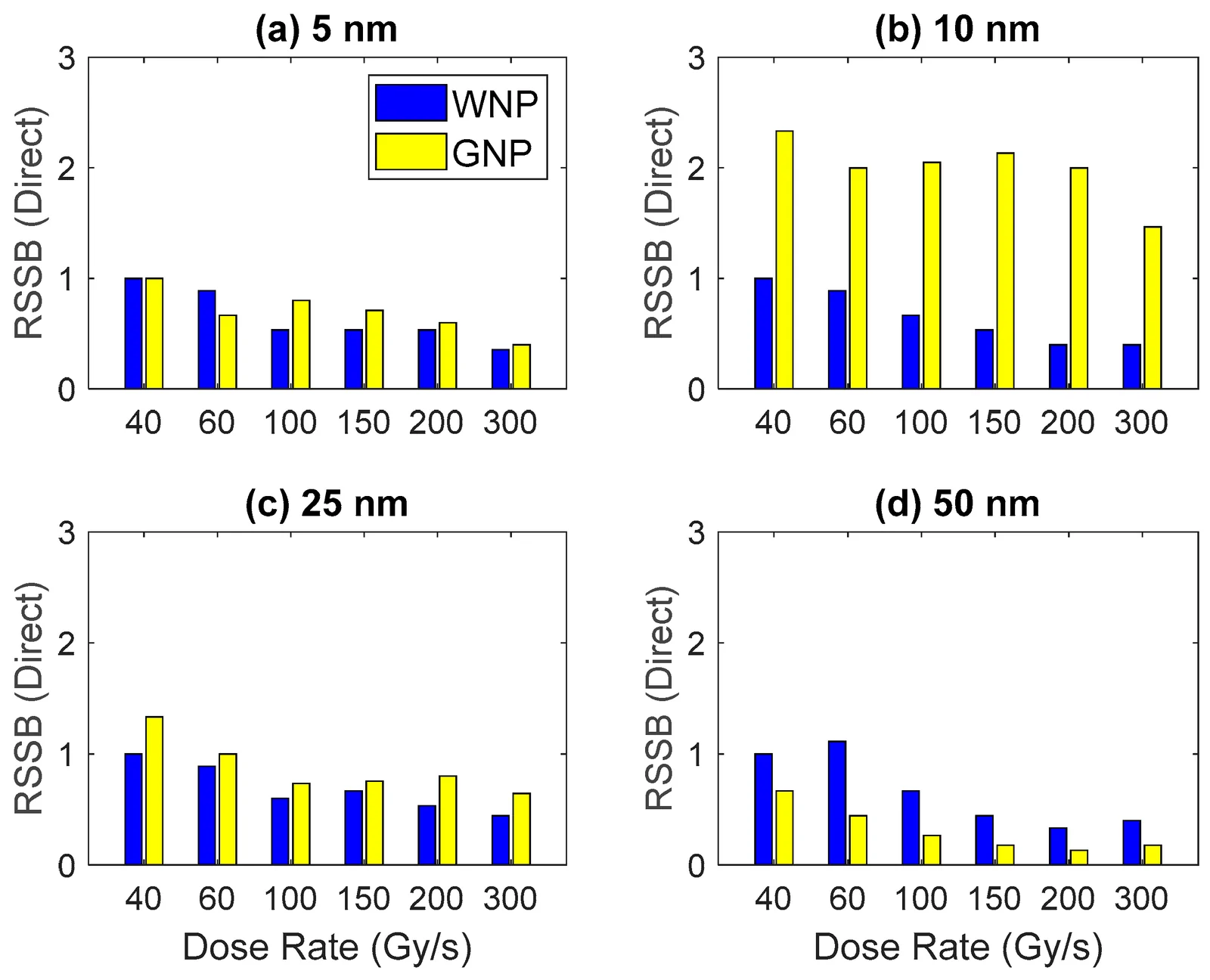
Variation in RSSBs resulting from direct DNA damage under UHDR irradiation (40, 60, 100, 150, 200, and 300 Gy/s) in the presence of WNP and GNP with diameters of (**a**) 5 nm, (**b**) 10 nm, (**c**) 25 nm, and (**d**) 50 nm, under the electron energy of 10 keV.

The influence of UHDRs (40, 60, 100, 150, 200, and 300 Gy/s) on RSSB was systematically investigated using GNPs with diameters of 5, 10, 25, and 50 nm. The simulations were performed using 10 keV and 1 MeV electrons to observe RSSBs resulting from direct and indirect DNA damage mechanisms. As shown in Figure 2, direct DNA damage-induced RSSB yields from 10 keV electrons decreased with increasing dose rate for WNPs with diameters of 5, 10, 25, and 50 nm, with normalized yields declining from 1.00 to 0.36, 1.00 to 0.40, 1.00 to 0.44, and 1.00 to 0.40, respectively. Similarly, the RSSBs for GNPs decreased in a dose rate-dependent manner, with values decreasing from 1.00 to 0.40, 2.33 to 1.47, 1.33 to 0.64, and 0.67 to 0.18, respectively. Nevertheless, the RSSB yields remained enhanced relative to those of WNPs for GNP diameters of 5, 10, and 25 nm. Among the GNP diameters investigated, the 10 nm GNP yielded the greatest enhancement in RSSBs, whereas the 50 nm GNP resulted in a substantial reduction in RSSBs.

**Figure 3:**
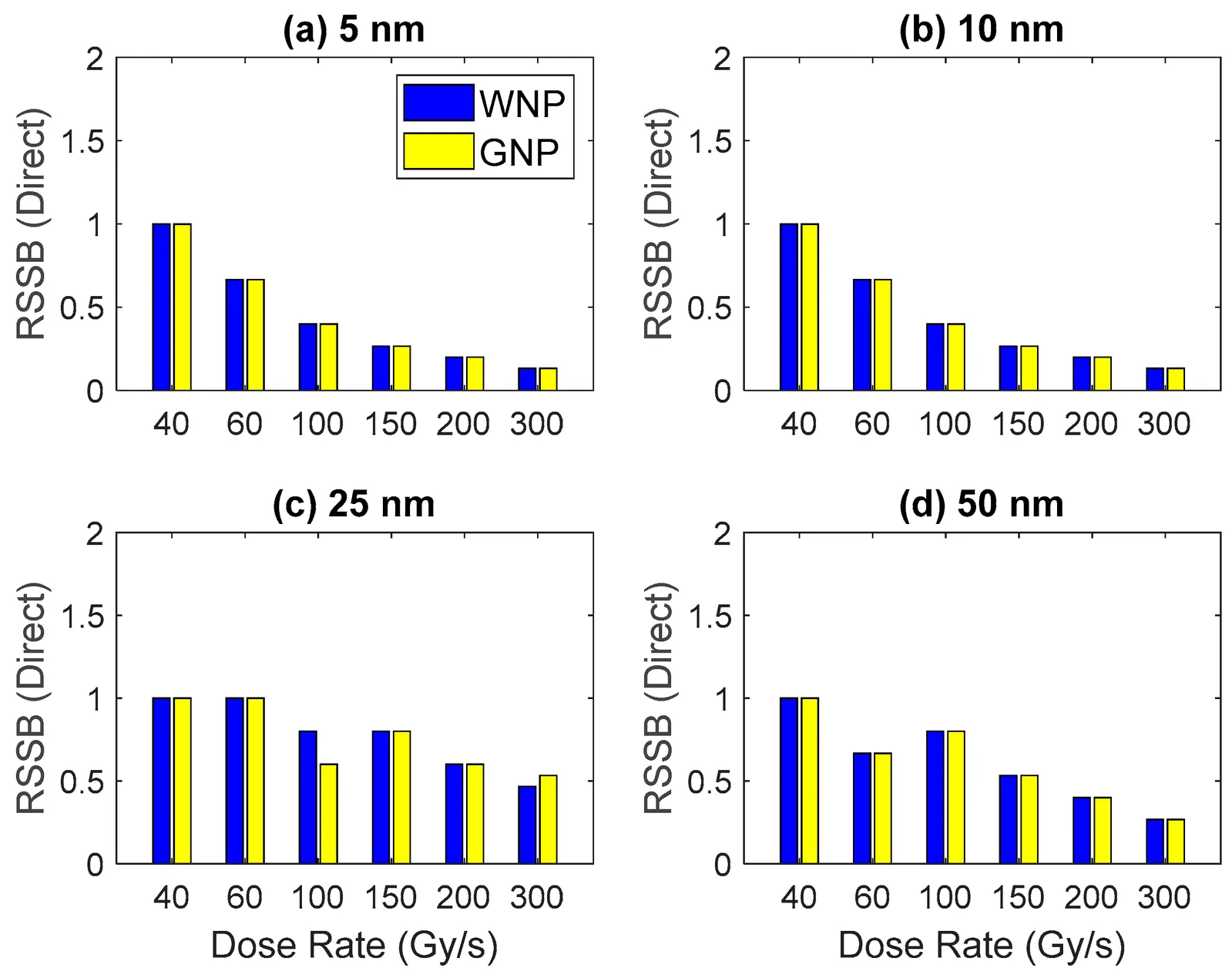
Variation in RSSBs resulting from direct DNA damage under UHDR irradiation (40, 60, 100, 150, 200, and 300 Gy/s) in the presence of WNP and GNP with diameters of (**a**) 5 nm, (**b**) 10 nm, (**c**) 25 nm, and (**d**) 50 nm, under the electron energy of 1 MeV.

For 1 MeV electron energy (Figure 3), direct DNA damage-induced RSSBs for both WNPs and GNPs decreased with increasing dose rate. The reduction was most pronounced for the 5 and 10 nm particles, with RSSB values decreasing from 1.00 at the lowest dose rate to 0.13 at the highest dose rate, whereas the corresponding values for the 25 and 50 nm particles decreased from 1.00 to 0.26. Unlike the observations at 10 keV, the presence of GNPs resulted in little change in RSSB production relative to WNPs.

**Figure 4:**
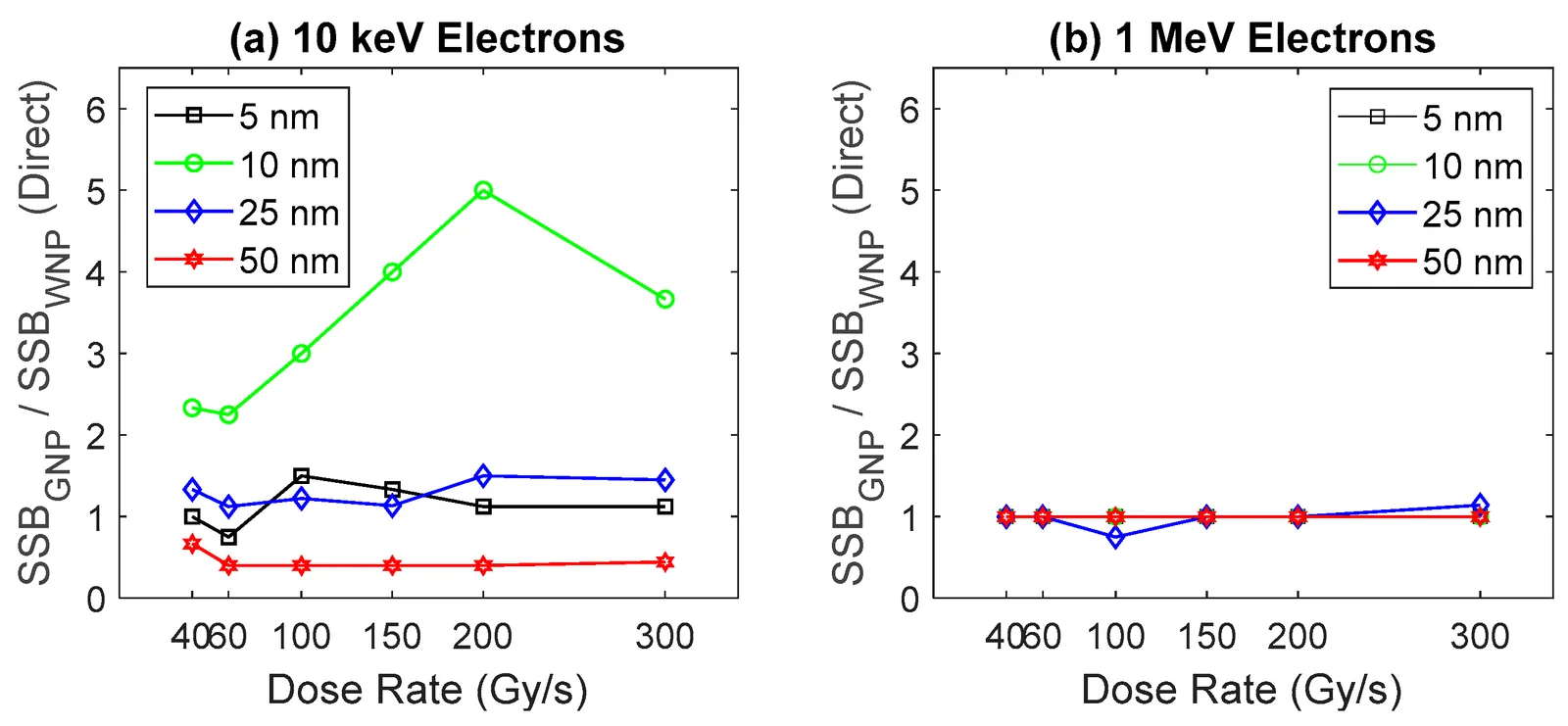
Ratio of direct DNA damage-induced SSB yields for GNPs of varying sizes relative to those for WNPs under UHDR irradiation at dose rates of 40, 60, 100, 150, 200, and 300 Gy/s for electron energies of (**a**) 10 keV and (**b**) 1 MeV.

The matched GNP/WNP enhancement ratios for direct DNA damage-induced SSBs are shown in Figure 4. For direct DNA damage-induced SSBs under 10 keV irradiation (Figure 4a), the 10 nm GNP produced the greatest enhancement among the particle sizes investigated, with the matched GNP/WNP enhancement ratio reaching a maximum of 5.00 at 200 Gy/s. In contrast, the 50 nm GNP produced enhancement ratios below unity across the investigated dose rates, indicating lower direct SSB yields than those obtained with the corresponding 50 nm WNP control. For 1 MeV electrons (Figure 4b), the enhancement ratios remained close to unity for most nanoparticle sizes and dose rates, indicating little GNP-specific enhancement of direct SSB production under these conditions.

**Figure 5:**
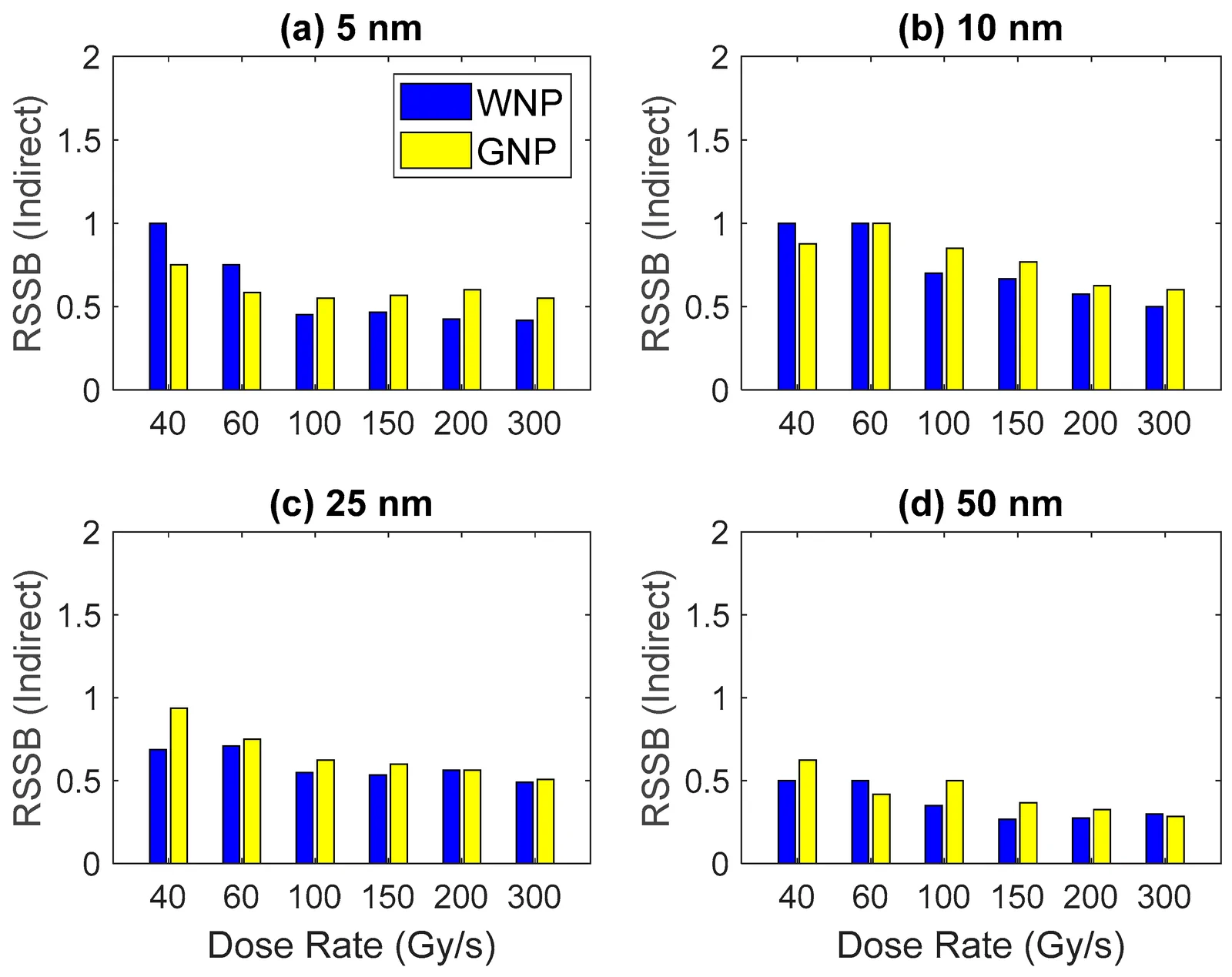
Variation in RSSBs resulting from indirect DNA damage under UHDR irradiation (40, 60, 100, 150, 200, and 300 Gy/s) in the presence of WNPs and GNPs with diameters of (**a**) 5 nm, (**b**) 10 nm, (**c**) 25 nm, and (**d**) 50 nm, under the electron energy of 10 keV.

Figure 5 illustrates the indirect DNA damage-induced RSSBs for 10 keV electrons. For both WNPs and GNPs, RSSB yields decreased moderately, from 1.00 to 0.42 for the 5 and 10 nm particles and from 1.00 to 0.51 for the 25 nm particles. However, the 50 nm particles exhibited a greater reduction, with yields decreasing from 0.50 to 0.30 for WNPs and from 0.63 to 0.28 for GNPs. Notably, the patterns and magnitudes of RSSB decrease were comparable for GNPs and WNPs, suggesting that GNPs did not produce an appreciable enhancement.

**Figure 6:**
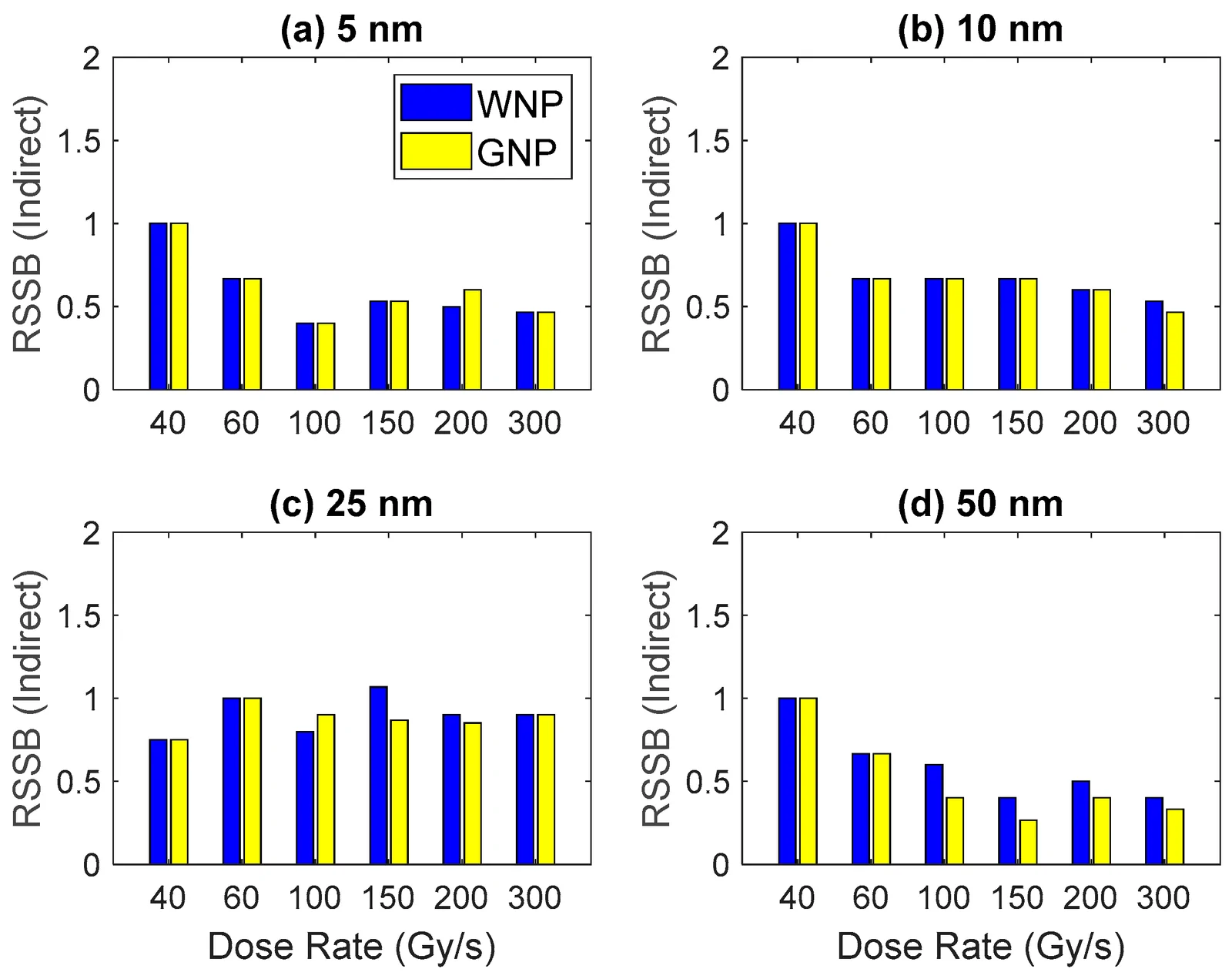
Variation in RSSBs resulting from indirect DNA damage under UHDR irradiation (40, 60, 100, 150, 200, and 300 Gy/s) in the presence of WNPs and GNPs with diameters of (**a**) 5 nm, (**b**) 10 nm, (**c**) 25 nm, and (**d**) 50 nm, under the electron energy of 1 MeV.

As shown in Figure 6, the indirect DNA damage-induced RSSBs generated by 1 MeV electrons followed a trend similar to that observed for 10 keV electrons, indicating that GNPs produced little enhancement in RSSB production compared with WNPs. For both WNPs and GNPs, only a modest decrease in RSSB yield was observed with increasing dose rate, with values declining from 1.0 to 0.4 for particle diameters of 5 and 10 nm, and from 1.0 to 0.27 for a particle diameter of 50 nm. For the 25 nm particles, a smaller decrease was observed, with RSSB yields ranging from 1.00 to 0.75 for WNPs and from 1.00 to 0.85 for GNPs.

**Figure 7:**
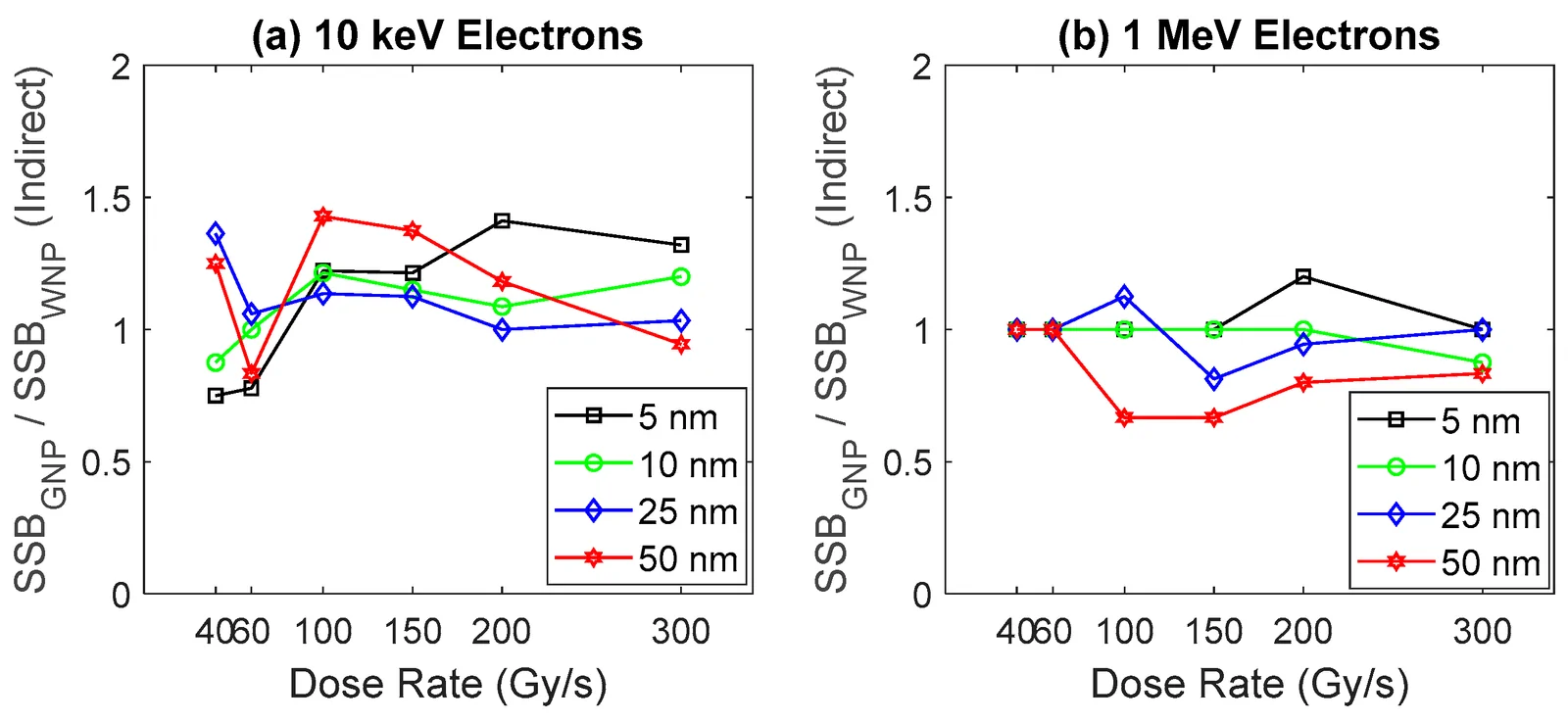
Ratio of indirect DNA damage-induced SSB yields for GNPs of varying sizes relative to those for WNPs under UHDR irradiation at dose rates of 40, 60, 100, 150, 200, and 300 Gy/s for electron energies of (**a**) 10 keV and (**b**) 1 MeV.

For indirect DNA damage-induced SSBs (Figure 7), the matched GNP/WNP enhancement ratios ranged from 0.75 to 1.43 for 10 keV electrons and from 0.67 to 1.20 for 1 MeV electrons. These values were substantially lower than the maximum enhancement observed for direct SSB induction, indicating that the GNP-specific enhancement was more pronounced for the direct damage mechanism under the conditions investigated. In particular, the 50 nm GNP under 1 MeV electron irradiation produced enhancement ratios below unity, indicating reduced indirect SSB yields relative to the corresponding WNP control.

### 3.2. Influence of GNP Radiosensitization on RDSBs Under UHDR Irradiation

**Figure 8:**
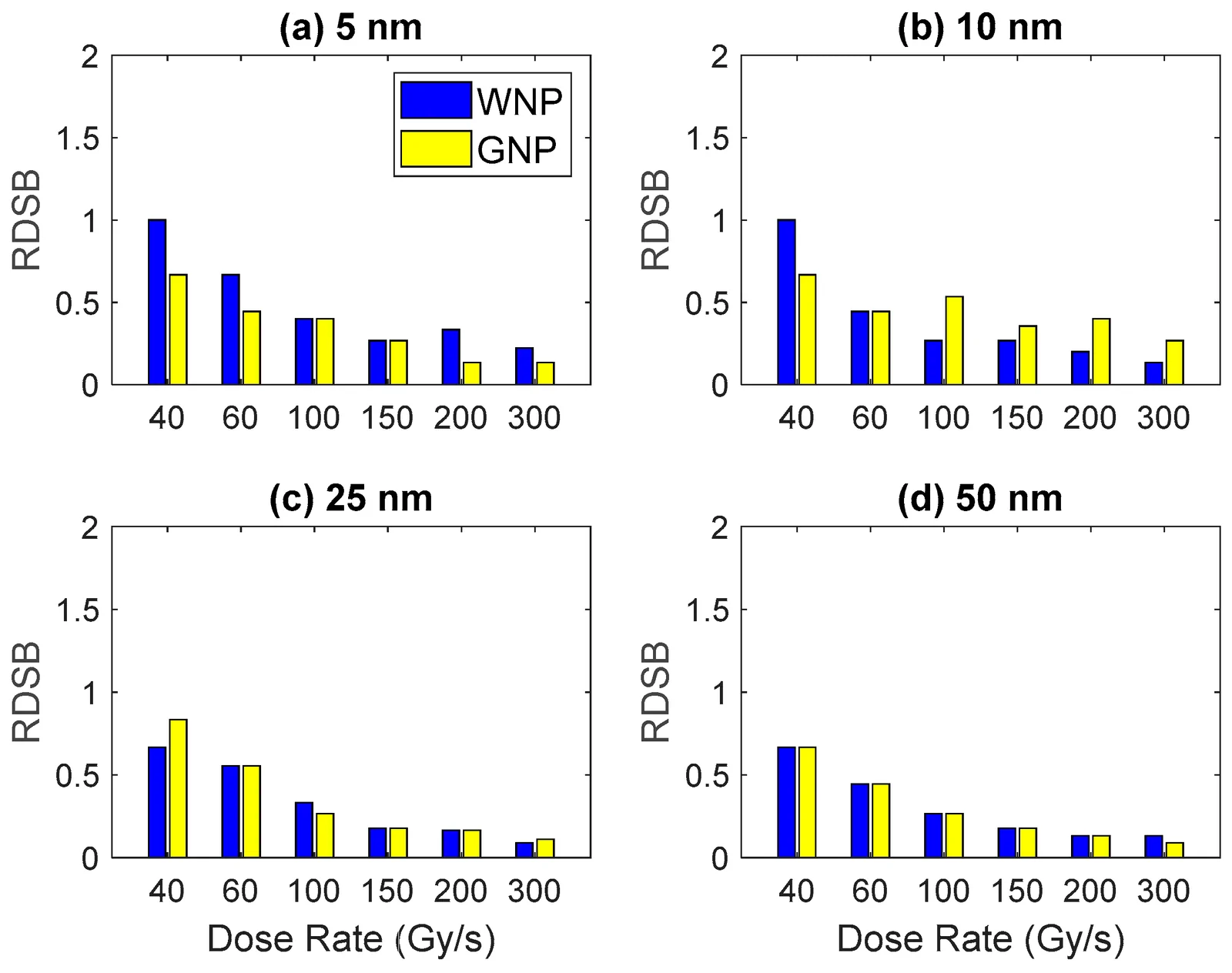
Variation in RDSB yields under UHDR irradiation (40, 60, 100, 150, 200, and 300 Gy/s) in the presence of WNPs and GNPs with diameters of (**a**) 5 nm, (**b**) 10 nm, (**c**) 25 nm, and (**d**) 50 nm at an electron energy of 10 keV.

Figure 8 shows the influence of UHDRs (40, 60, 100, 150, 200, and 300 Gy/s) at 10 keV on RDSB yields in the presence of GNPs with diameters of 5, 10, 25, and 50 nm. For the 5 nm WNP, the RDSB yield decreased from 1.00 to 0.22 with increasing dose rate. However, the decrease in RDSB yield was more pronounced for GNP than for WNP, with the yield declining from 0.67 to 0.13. In contrast, an enhancement in RDSB yield was observed for the 10 nm GNP at dose rates of 100–300 Gy/s relative to WNP, with yields decreasing from 0.54 to 0.27 for GNP and from 0.26 to 0.13 for WNP. For 25 and 50 nm particles, increasing the dose rate reduced RDSB yields for both WNPs and GNPs. Despite this trend, the presence of GNPs had little influence on RDSB production relative to WNPs.

**Figure 9:**
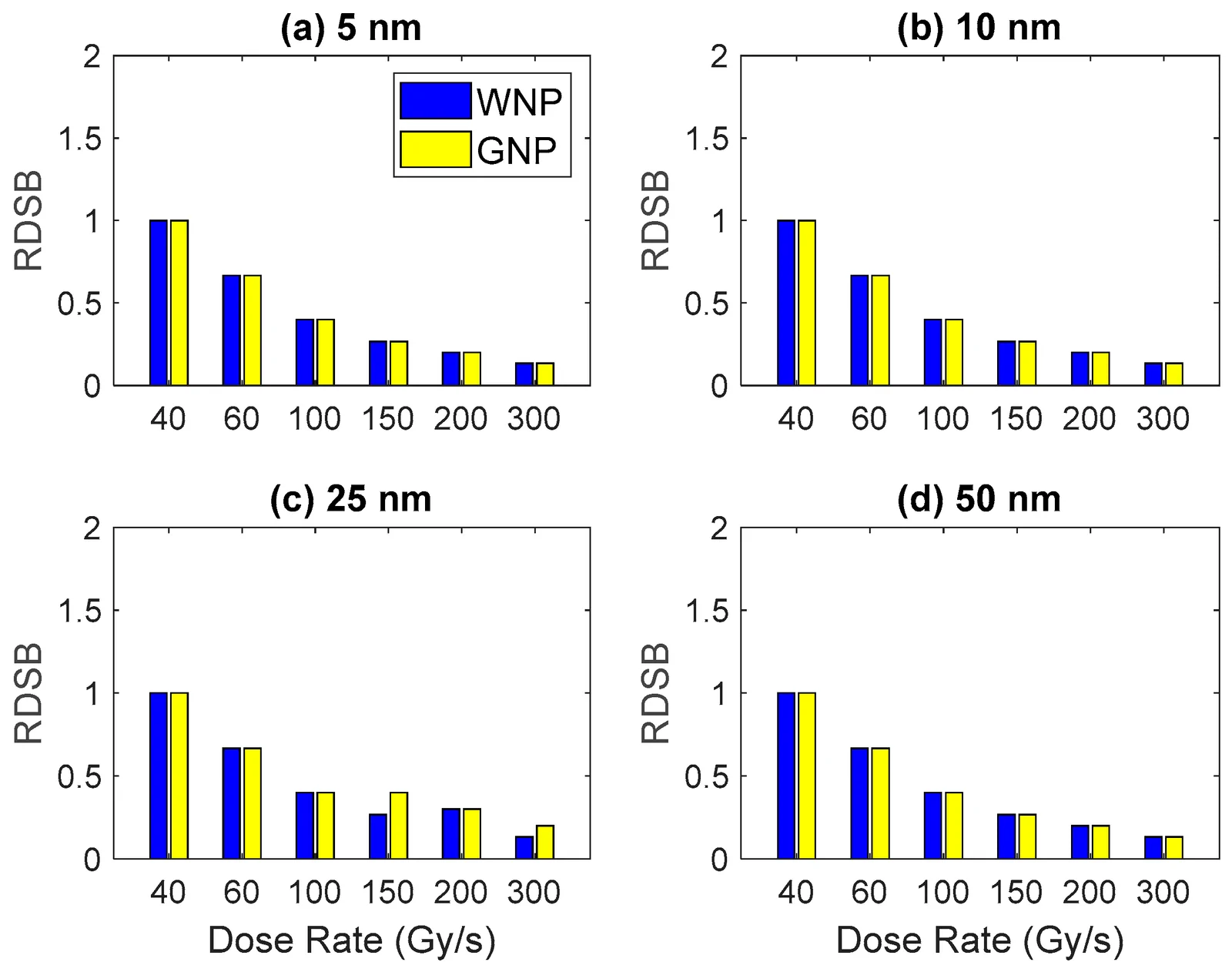
Variation in RDSB yields under UHDR irradiation (40, 60, 100, 150, 200, and 300 Gy/s) in the presence of WNPs and GNPs with diameters of (**a**) 5 nm, (**b**) 10 nm, (**c**) 25 nm, and (**d**) 50 nm at an electron energy of 1 MeV.

In contrast to the 10 keV case, as shown in Figure 9, the RDSB yield at 1 MeV showed a similar dose-rate dependence for both WNPs and GNPs, decreasing from 1.00 to 0.13 across all particle diameters (5, 10, 25, and 50 nm). The RDSB yields were comparable between GNPs and WNPs across the investigated particle sizes, indicating that GNP size had no measurable influence on RDSB formation.

**Figure 10:**
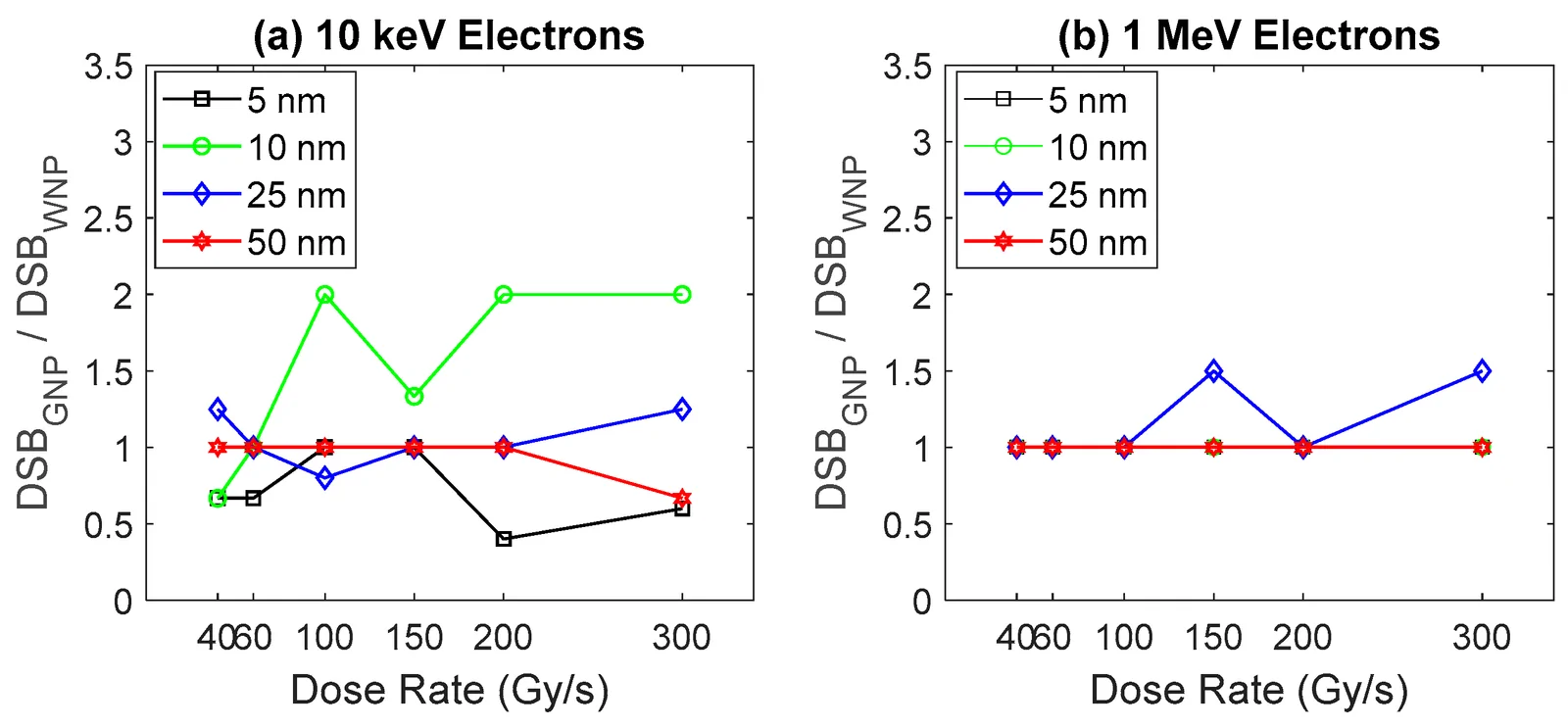
Ratio of DSB yields for GNPs of varying sizes relative to those for WNPs under UHDR irradiation at dose rates of 40, 60, 100, 150, 200, and 300 Gy/s for electron energies of (**a**) 10 keV and (**b**) 1 MeV.

Figure 10 shows the matched GNP/WNP enhancement ratios for DSB yields. For 10 keV electrons (Figure 10a), the 10 nm GNP produced enhancement ratios ranging from 1.33 to 2.00 at dose rates of 100–300 Gy/s, demonstrating greater DSB induction than the corresponding 10 nm WNP control. In contrast, for 1 MeV electrons (Figure 10b), the enhancement ratios remained closer to unity across most nanoparticle sizes and dose rates, indicating substantially weaker GNP-specific enhancement of DSB production.

Table 1 presents the absolute SSB and DSB yields per Gy for different sizes of GNPs and WNPs under various UHDR conditions for 10 keV and 1 MeV electron energies.

## 4. Discussion

### 4.1. Dose Rate Effects on RSSB, RDSB, SSB, and DSB Yields

The results revealed a dose rate-dependent decrease in both direct and indirect DNA damage-induced RSSBs for 1 MeV electrons, consistent with previous reports [76,77]. ROS production in the vicinity (10 nm) of GNPs was highest at the lower dose rate (40 Gy/s), which is consistent with previous findings showing that ROS (H_2_O_2_) production decreases as the mean dose rate of UHDR irradiation increases [78]. The reduced ROS production at higher dose rates, through rapid depletion of local oxygen concentration and radical–radical recombination, may contribute to the decreased formation of indirect DNA damage-induced RSSBs under high-dose-rate electron beams [79]. In addition, it has been proposed that the abundance of low-energy secondary electrons generated under UHDR irradiation enhances dissociative electron attachment (DEA) [80], which may further contribute to the reduced formation of direct DNA damage-induced RSSBs. It should also be noted that the RDSBs for 1 MeV electrons exhibited a trend similar to that observed for the RSSBs, decreasing with increasing dose rate, as shown in Figure 7. This observation is expected because DSBs are scored based on the occurrence of two SSBs within 10 base pairs of each other. When the results for GNP were compared with those for WNP, no enhancement in RSSBs and RDSBs was observed with GNP. This can be attributed to the inability of high-energy megavoltage beams, such as 1 MeV electron beams, to generate significant local electronic cascades in high-Z materials like GNPs [81]. In addition, direct DNA damage-induced SSB and DSB yields in the presence of 10 nm GNPs increased under high UHDR conditions (100–300 Gy/s) at an electron energy of 10 keV, whereas no appreciable dose rate-dependent changes were observed at 1 MeV.

Although low-energy electrons (10 keV) also exhibited dose rate-dependent decreases in both RSSBs and RDSBs, these decreases were less pronounced than those observed for high-energy electrons (1 MeV). The weaker dose-rate dependence observed for low-energy electrons (10 keV) may be attributed to their shorter track lengths and reduced penetration depth, which are expected to generate a greater number of low-energy secondary electrons and, consequently, more ROSs in the vicinity of DNA, thereby partially offsetting the reduction in SSBs and DSBs at lower dose rates.

It should be emphasized that the 10 keV and 1 MeV electron energies considered in this study represent simplified monoenergetic model conditions rather than the broad electron energy spectrum encountered in clinical FLASH irradiation. In particular, the 10 keV condition was selected to investigate the mechanistic contribution of low-energy electron interactions to GNP-mediated secondary-electron production and DNA damage at the nanoscale. Therefore, the enhanced radiosensitization observed at 10 keV should be interpreted as a mechanistic finding under the model conditions investigated and should not be directly extrapolated to the response of GNPs under a clinical FLASH electron beam. Future studies incorporating realistic electron energy spectra are needed to evaluate the extent to which these nanoscale effects translate to clinically relevant irradiation conditions.

### 4.2. GNP Size on RSSB and RDSB

For 10 keV electrons, direct DNA damage-induced RSSBs increased as the GNP size increased from 5 nm, reached a peak at 10 nm, and subsequently decreased with further increases in GNP size. A maximum 5-fold increase in SSB yields and 2-fold increase in DSB yields were observed in the presence of GNPs compared with WNPs. For smaller GNPs, a greater proportion of secondary electrons can escape the GNP and reach the surrounding medium, thereby increasing the probability of interactions with DNA and contributing to enhanced DNA damage. However, with large GNPs, low-energy secondary electrons are reabsorbed or scattered internally [82,83,84], reducing the RSSBs. As shown in Figure 2b, the enhanced direct DNA damage-induced RSSBs observed for the 10 nm GNP are likely attributable to the abundant secondary electrons generated by the GNP. These secondary electrons also enhance ROS production, contributing to the increased yield of indirect RSSBs (Figure 5, Figure 6 and Figure 7) and the increase in RDSBs (Figure 8, Figure 9 and Figure 10). At an electron energy of 1 MeV, RSSB and RDSB yields were largely independent of GNP size. The diameter of GNP which yields increased RSSB and RDSB differs from the energy of the electron used for the simulations. These findings underscore the need for further investigation into the influence of GNP size on radiosensitization, with particular emphasis on determining how nanoparticle size and irradiation conditions influence SSB and DSB induction under UHDR irradiation. The four GNP diameters investigated (5, 10, 25, and 50 nm) represent discrete model conditions and do not constitute a continuous optimization of nanoparticle size; therefore, intermediate or larger/smaller particle sizes may exhibit different enhancement behaviour.

Importantly, the greater enhancement observed for the 10 nm GNP should be interpreted within the specific irradiation conditions investigated in this study. The 10 nm GNP produced the greatest DNA-damage enhancement among the particle sizes tested under the 10 keV monoenergetic, single-pulse condition, whereas a comparable size-dependent enhancement was not observed at 1 MeV. Therefore, the present results do not establish 10 nm as a universally optimal GNP size for FLASH radiotherapy. The optimal nanoparticle size may depend on electron energy, irradiation conditions, nanoparticle distribution, and other physical and biological factors.

It should be noted that the present simulations considered a single isolated GNP positioned at a fixed distance from the DNA model. In biological environments, GNPs may form aggregates, resulting in more complex nanoparticle geometries than the isolated-particle configuration considered here. Aggregation may alter the production, escape, and spatial distribution of low-energy secondary electrons and radiation-induced ROS in the vicinity of DNA. In particular, secondary electrons generated within an aggregate may undergo additional scattering or reabsorption by neighbouring GNPs before reaching the surrounding aqueous medium, potentially reducing the local DNA-damage enhancement predicted for an isolated GNP. At the same time, interactions among closely spaced GNPs may modify local energy deposition and ROS distributions, making the magnitude of this effect dependent on aggregate size, morphology, and proximity to DNA. Therefore, the enhancement ratios obtained in the present study should be interpreted as predictions for the idealized single-GNP geometry investigated rather than as direct estimates of DNA damage in biologically aggregated GNP systems. Future simulations incorporating multiple GNPs and physiologically relevant aggregation geometries are warranted to determine how nanoparticle aggregation modifies GNP-mediated radiosensitization under UHDR irradiation.

The fixed 10 nm GNP–DNA separation used in the present simulations represents another idealization of the nanoparticle geometry. Because low-energy secondary electrons and short-lived reactive species have limited ranges in the surrounding medium, the magnitude of DNA-damage enhancement is expected to depend on the distance between the GNP and DNA. Consequently, the enhancement factors reported here apply specifically to the 10 nm separation investigated and should not be assumed to remain unchanged for other intracellular GNP–DNA distances. A systematic evaluation of GNP–DNA separation would be valuable in future studies.

The calculated SSB and DSB yields also depend on the DNA-damage scoring criteria adopted in the simulations. Direct SSBs were scored using an energy-deposition threshold of 17.5 eV, indirect SSBs were assigned with a probability of 40% following OH• interaction with the DNA backbone, and DSBs were defined as two SSBs occurring on opposite strands within 10 base pairs. Although these criteria are based on previously established Geant4-DNA approaches [74,75], alternative scoring thresholds or probabilities may alter the absolute predicted DNA-damage yields. The present quantitative results should therefore be interpreted within the scoring framework employed in this study.

It should be noted that UHDR irradiation reduced the yields of RSSBs and RDSBs, consistent with the normal tissue–sparing effect associated with FLASH radiotherapy. Nevertheless, the increased DNA damage observed in the presence of GNPs suggests that GNPs located in close proximity to cellular DNA can locally enhance the induction of both SSBs and DSBs, even under UHDR irradiation conditions.

## 5. Conclusions

Monte Carlo (MC) simulations using Geant4-DNA were performed to quantify DNA SSBs and DSBs under UHDR electron-beam irradiation, simulating FLASH radiotherapy conditions. To investigate the radiosensitization effect of GNPs, the yields of RSSBs and RDSBs for GNPs with diameters ranging from 5 to 50 nm were compared with those obtained using WNPs. The results demonstrated a dose rate-dependent reduction in both direct and indirect DNA damage-induced RSSBs and RDSBs under electron-beam irradiation. This dose-dependent reduction was more pronounced for high-energy electrons (1 MeV) compared with low-energy electrons (10 keV).

Among the GNP sizes investigated (5, 10, 25, and 50 nm), the 10 nm GNP produced the greatest DNA-damage enhancement under the 10 keV monoenergetic, single-pulse irradiation condition. However, a comparable size-dependent enhancement was not observed at 1 MeV. Therefore, the present findings should not be interpreted as identifying 10 nm as a universally optimal GNP size; rather, they demonstrate that GNP-mediated radiosensitization depends strongly on nanoparticle size and electron energy under the specific irradiation conditions investigated. Although the MC simulations employed in this study represent a simplified model of complex biological environments, with limitations arising from single-pulse irradiation and idealized conditions, they provide valuable mechanistic insights into GNP-mediated radiosensitization at UHDR.

The use of isolated GNPs and a single-pulse irradiation model represents a limitation of the present study. In biological environments, GNP aggregation may alter secondary-electron escape and the spatial distribution of radiation-induced ROS, thereby changing the magnitude of local DNA-damage enhancement relative to that predicted for an isolated GNP. Therefore, the present results should be interpreted within the single-particle geometry investigated. Future studies incorporating multiple GNPs, physiologically relevant aggregation geometries, and more realistic irradiation conditions are warranted to further evaluate GNP-mediated radiosensitization under UHDR irradiation.

## Figures and Tables

**Table 1:** Absolute SSB and DSB yields (Counts/Gy): Water nanoparticle (W) and gold nanoparticle (G).

		**SSB (Direct) with 10 keV Electrons**								**SSB (Direct) with 1 MeV Electrons**							
**Particle Size**		**5 nm**		**10 nm**		**25 nm**		**50 nm**		**5 nm**		**10 nm**		**25 nm**		**50 nm**	
**Particle Type**		**W**	**G**	**W**	**G**	**W**	**G**	**W**	**G**	**W**	**G**	**W**	**G**	**W**	**G**	**W**	**G**
**Dose** **rate** **(Gy/s)**	40	45	45	45	105	45	60	45	30	30	30	30	30	30	30	30	30
	60	40	30	40	90	40	45	50	20	20	20	20	20	30	30	20	20
	100	24	36	30	90	27	33	30	12	12	12	12	12	24	18	24	24
	150	24	32	24	96	30	34	20	8	8	8	8	8	24	24	16	16
	200	24	27	18	90	24	36	15	6	6	6	6	6	18	18	12	12
	300	16	18	18	66	20	29	18	8	4	4	4	4	14	16	8	8
		**SSB (Indirect) with 10 keV Electrons**								**SSB (Indirect) with 1 MeV Electrons**							
**Particle Size**		**5 nm**		**10 nm**		**25 nm**		**50 nm**		**5 nm**		**10 nm**		**25 nm**		**50 nm**	
**Particle Type**		**W**	**G**	**W**	**G**	**W**	**G**	**W**	**G**	**W**	**G**	**W**	**G**	**W**	**G**	**W**	**G**
**Dose** **rate** **(Gy/s)**	40	120	90	120	105	83	113	60	75	60	60	60	60	45	45	60	60
	60	90	70	120	120	85	90	60	50	40	40	40	40	60	60	40	40
	100	54	66	84	102	66	75	42	60	24	24	39	39	48	54	36	24
	150	56	68	80	92	64	72	32	44	32	32	40	40	64	52	24	16
	200	51	72	69	75	68	68	33	39	30	36	36	36	54	51	30	24
	300	50	66	60	72	59	61	36	34	28	28	32	28	54	54	24	20
		**DSB with 10 keV Electrons**								**DSB with 1 MeV Electrons**							
**Particle Size**		**5 nm**		**10 nm**		**25 nm**		**50 nm**		**5 nm**		**10 nm**		**25 nm**		**50 nm**	
**Particle Type**		**W**	**G**	**W**	**G**	**W**	**G**	**W**	**G**	**W**	**G**	**W**	**G**	**W**	**G**	**W**	**G**
**Dose** **rate** **(Gy/s)**	40	45	30	45	30	30	38	30	30	30	30	30	30	30	30	30	30
	60	30	20	20	20	25	25	20	20	20	20	20	20	20	20	20	20
	100	18	18	12	24	15	12	12	12	12	12	12	12	12	12	12	12
	150	12	12	12	16	8	8	8	8	8	8	8	8	8	12	8	8
	200	15	6	9	18	8	8	6	6	6	6	6	6	9	9	6	6
	300	10	6	6	12	4	5	6	4	4	4	4	4	4	6	4	4

## Data Availability

The raw data supporting the conclusions of this article will be made available by the authors on request.

## Abbreviations

The following abbreviations are used in this manuscript:

Abbreviation	Full Term
UHDR	Ultrahigh Dose Rate
FLASH-RT	FLASH Radiotherapy
CONV-RT	Conventional Radiotherapy
GNP	Gold Nanoparticle
WNP	Water Nanoparticle
ROS	Reactive Oxygen Species
DNA	Deoxyribonucleic Acid
MC	Monte Carlo
Geant4-DNA	Geant4-DNA Simulation Toolkit
SSB	Single-Strand Break
DSB	Double-Strand Break
RSSB	Relative Single-Strand Break
RDSB	Relative Double-Strand Break
LINAC	Linear Accelerator
IGRT	Image-Guided Radiotherapy
VMAT	Volumetric Modulated Arc Therapy
ENM	Engineered Nanomaterial
ISO	International Organization for Standardization
OH•	Hydroxyl Radical
DEA	Dissociative Electron Attachment
H_2_O_2_	Hydrogen Peroxide
e^−^aq	Aqueous Electron (Hydrated Electron)
H_3_O^+^	Hydronium Ion
bp	Base Pair
keV	Kiloelectron Volt
MeV	Megaelectron Volt
nm	Nanometre
μm	Micrometre
CERN	European Organization for Nuclear Research
